# From scaling up to sustainability in HIV: potential lessons for moving forward

**DOI:** 10.1186/1744-8603-9-57

**Published:** 2013-11-07

**Authors:** Lisa R Hirschhorn, Julie R Talbot, Alexander C Irwin, Maria A May, Nayana Dhavan, Robert Shady, Andrew L Ellner, Rebecca L Weintraub

**Affiliations:** 1 Department of Global Health and Social Medicine, Harvard Medical School, 641 Huntington Avenue, Boston, MA, USA; 2 Partners in Health, 888 Commonwealth Avenue, Boston, MA, USA; 3 The Global Health Delivery Project at Harvard University, 75 Francis Street, Boston, MA, USA; 4 Department of Global Health Equity, Brigham and Women’s Hospital, 75 Francis Street, Boston, MA, USA

**Keywords:** Scale-up, Sustainability, Large-scale, HIV, Literature review, Framework, Public health, Care delivery

## Abstract

**Background:**

In 30 years of experience in responding to the HIV epidemic, critical decisions and program characteristics for successful scale-up have been studied. Now leaders face a new challenge: sustaining large-scale HIV prevention programs. Implementers, funders, and the communities served need to assess what strategies and practices of scaling up are also relevant for sustaining delivery at scale.

**Methods:**

We reviewed white and gray literature to identify domains central to scaling-up programs and reviewed HIV case studies to identify how these domains might relate to sustaining delivery at scale.

**Results:**

We found 10 domains identified as important for successfully scaling up programs that have potential relevance for sustaining delivery at scale: fiscal support; political support; community involvement, integration, buy-in, and depth; partnerships; balancing flexibility/adaptability and standardization; supportive policy, regulatory, and legal environment; building and sustaining strong organizational capacity; transferring ownership; decentralization; and ongoing focus on sustainability. We identified one additional potential domain important for programs sustaining delivery at scale: emphasizing equity.

**Conclusions:**

Today, the public and private sector are examining their ability to generate value for populations. All stakeholders are aiming to stem the tide of the HIV epidemic. Implementers need a framework to guide the evolution of their strategies and management practices. Greater research is needed to refine the domains for policy and program implementers working to sustain HIV program delivery at scale.

## Background

In the past decade, global public health has seen advances in access to HIV prevention, care, and treatment, enabled by unprecedented levels of new financing. The paradigm began as an emergency response catalyzed by new institutions including the Global Fund to Fight AIDS, Tuberculosis and Malaria and the President’s Emergency Plan for AIDS Relief (PEPFAR). Organizations were encouraged to scale up rapidly to test, treat, and prevent as many new HIV/AIDS infections as possible to halt the epidemic. This work has led to dramatic declines in HIV-related deaths and in new infections [1].

The remarkable success of these programs has resulted in the transformation of HIV into a chronic disease, requiring lifetime commitment for treatment. However, the absence of a proven vaccine or cure demands that prevention and treatment programs must continue to deliver at scale for the foreseeable future [1].

A number of HIV prevention and treatment programs that have achieved scale are focused on understanding how to improve and sustain service delivery while continuing to adapt to scientific advances [2,3]. Some approaches have included integrating services into existing systems of care, such as antenatal and TB clinics, as well as task sharing and other approaches to address common challenges [4,5]. In addition, the call for increased national ownership creates an urgent need to identify how to migrate programs that have scaled up partially or largely outside of the public sector [6].

In 2010, we investigated how HIV prevention programs had achieved delivery at scale. We documented programs in detail, compiling a collection of case studies that represent a rich source of data and describe the operations and management complexities of these programs.^a^ In addition, the cases capture the broader context underlying the programs, such as the political situation, epidemiology, and demography. Simultaneously, we also explored the gray and white literature for frameworks that could help us elucidate the programs’ success and challenges in their work to sustain scale. However, while a number of frameworks for program directors, policy makers, and implementers have been developed to help navigate this path from innovation to scale up [7-11], we were not able to identify a similar wealth of work on how to sustain delivery at scale.

In this paper, we synthesize our findings from this framework review into domains recognized as integral for scaling up programs. We then explore the potential relevance of these domains to sustaining delivery at scale using the prevention case studies and additional areas that might be added to a sustaining delivery at scale framework. Our results identify a number of areas where more work is needed to further identify and validate the core domains relevant to sustaining delivery at scale for HIV programs. However, we believe that this work is an important initial step in developing a framework to understand how HIV and other programs can sustain efforts to provide effective and efficient services at scale over time in an ever-changing environment.

## Methods

### Case studies

We researched and produced 12 case studies (from 11 programs) on HIV prevention published by Harvard Business Publishing (see Table 1 for list of case studies) [12]. Cases were developed using the Harvard Business School case method describing the factors (internal and external) that contributed to the relative success or failure. This method does not attempt to isolate any individual factor from the entire system, but assumes dependence of many forces within the program and attempts to understand it in a way that acknowledges those interrelations.

**Table 1:** Global Health Delivery HIV case, VMMC case studies*

**Case title**	**Region**	**Primary sector**	**Type of scale-up**	**Storyline**
Treating HIV in Kenya: the Academic Model for the Prevention and Treatment of HIV/AIDS (AMPATH)	East Africa	Private	Quantitative, functional	Academic partnership between Indiana U. and Kenyan U. leads to primary care and soon large-scale HIV/AIDS treatment in Kenya.
The AIDS Support Organization (TASO) of Uganda	East Africa	Private	Quantitative	HIV/AIDS support organization in Uganda scales up, offers treatment, and quickly expands number of sites/clinics offering services at the request of the government.
Botswana’s Program for Preventing Mother-to-Child HIV Transmission	Southern Africa	Public	Quantitative	Botswana implements a national PMTCT program and finds unforeseen challenges.
Iran's Triangular Clinic	Middle East	Public	Quantitative	An innovative HIV/AIDS harm reduction clinic in Kermanshah Province, Iran, overcomes cultural hurdles to offer support, prevention, and treatment and is replicated in prisons before being scaled up nationally.
HIV in Thailand: The 100% Condom Program	Southeast Asia	Public	Quantitative, political	A regional Ministry of Health director implements a policy mandating that commercial sex workers use condoms and gets all regional stakeholders on board before considering scale-up.
HIV Voluntary Counseling and Testing in Hinche, Haiti	Caribbean	Private	Quantitative	A nongovernmental organization (NGO) offering health care in one area of Haiti is asked to help rejuvenate voluntary counseling and services at a poorly functioning government site in another area of the country.
HIV Care in Rwanda	East Africa	Private	Quantitative, functional	The Rwandan Ministry of Health invites an NGO to assume responsibility for health care services and create an HIV program in two districts before considering national scale-up.
HIV/AIDS in Brazil: Delivering Prevention in a Decentralized Health System	South America	Public	Quantitative, political	Brazil scales up its response to HIV via a human rights framework, cooperating with civil society.
loveLife**: Preventing HIV Among South African Youth	Southern Africa	Private	Quantitative, organizational	The NGO loveLife scales up over time to prevent HIV among South African youth before losing one-third of its operating budget.
loveLife**: Transitions After 2005	Southern Africa	Private	Organizational	loveLife managers downsize and secure additional government funding to sustain the NGO.
The Avahan India AIDS Initiative: Managing Targeted HIV Prevention at Scale	South Asia	Private	Quantitative, political	The Avahan Indian AIDS Initiative (Avahan), an HIV prevention delivery program of the Bill & Melinda Gates Foundation, implements large-scale intervention strategies using a noteworthy structure, execution style, and management system.
HIV Prevention in Maharashtra, India	South Asia	Private	Quantitative, functional, organizational	Muslim Samaj Prabodhan Va Shikshan Sanstha (MSPSS), a grantee NGO with funding from Avahan, delivers high-value, comprehensive HIV preventive services to a high-risk population and must determine how to preserve the value of the program as it prepares to transition the program to government ownership.

*The full cases are available via http://www.ghdonline.org/cases.

**loveLife program was covered in two cases but analyzed as one for the purposes of this paper.

### Developing domains for delivery at scale

We conducted a review of recent published public health and development literature and related reports in the public domain to determine how scale is discussed and to identify existing theoretical models and frameworks for scaling up and sustaining delivery at scale. Potential sources were identified by a literature search of medical, public health, and general scholarly databases (PubMed, Google Scholar) using various combinations of the terms in Table 2.

**Table 2:** Search terms used for literature review

		
• Sustain	• Prevention	• Scaling up framework
• Scale	• HIV/AIDS	• Scale up
• Framework	• Transition	• Health and program or public health
• Sustainability	• Scaling up	• Health intervention
• Large scale	• Health	• Strategy

Our search yielded over 1,000 publications. Two authors (LRH, AI) reviewed the abstracts to identify potentially relevant articles, reports, and chapters for full review. We then reviewed bibliographies from identified publications for additional resources. In addition, we contacted experts in the fields of scaling up and international program capacity building for additional sources and reviewed the websites of major donors and international nongovernmental organizations (NGOs) involved in public health scale-up focusing on HIV and related fields. This process identified a total of 165 articles or other literature that described either scaling-up frameworks or programs that had achieved delivery at scale. From these we selected 15 sources that described frameworks and explicit characteristics or domains as important to scaling up successfully.

We then reviewed the 15 identified sources to: (1) identify differences and shared themes and concepts around scaling up; and (2) explore whether a synthesis of the frameworks was possible to develop a core set of domains. To reach these goals, each source was read by at least three team members to extract and code themes using modified grounded theory. An iterative group process was then used to merge the themes into domains. Domains were retained only if the themes they summarized were identified in three or more of the sources. Specific definitions for these domains were drawn from the primary sources. Any conflicting views or debates in the reviewed sources were represented in the definitions. The two authors who had extracted all 15 sources then reviewed the domains for consistency, and any differences in opinion were discussed with the remaining authors to develop final consensus.

#### ***Expert consultation***

In July 2010, we convened experts in the fields of scaling up HIV programs and health systems strengthening and research to discuss challenges and possible frameworks for sustaining delivery at scale [13]. The consolidated domains were presented to this group and refined based on feedback.

### Use of case studies for exploring sustaining delivery at scale domains

As noted, because none of the identified sources described frameworks or theoretical models focused primarily on sustaining delivery at scale (although some contained implicit or explicit discussions related to aspects of sustainability), we used the case studies to begin to explore the applicability of the scale-up domains for programs focused on sustaining delivery at scale. The mapping of the domains to the cases was also desigend to identify additional domains that might be relevant to sustainability.

## Results

### Defining “scale” and “scaling up”

We found that the definition of the term “scale” in the literature has expanded and evolved in the last 30 years, without an accepted and widely utilized version identified [10]. Earlier definitions of scale are narrow, reflecting quantitative measures of program scope, such as number of sites covered or beneficiaries served [14,15]. In more recent literature, most scholars view these definitions as insufficient. The CORE group, a collaboration of health professionals from international development NGOs, has proposed a broader conception of “scale”, defined as “widespread achievement of impact at affordable cost”, with “impact” serving as a function of coverage, effectiveness, efficiency, local ownership promoting sustainability, and equity [15]. CORE’s definition closely reflects those Coburn proposed [14] in the educational arena. Coburn’s framework included sustainability, spread, and shift in ownership, along with a fourth dimension: deep and consequential change.

Similar diversity is found in the scope of what “scaling up” entails. In a literature review on scaling up, Mangham and Hanson [10] argue, “scaling up is primarily used to describe the ambition or process of expanding the coverage of health interventions, though the term has also referred to increasing the financial, human, and capital resources required to expand coverage.” Although “scaling up” is still primarily employed to describe geographical reach, Uvin and Miller’s 1996 article, “Paths to Scaling Up”, sparked a more sophisticated dialogue by including dimensions beyond quantitative indicators: in addition to quantitative (geographic spread or expansion in size) scale up, they define “functional” scale-up as expanding the scope of activity; “political” scale-up as influencing the political process; and “organizational” scale-up as enhancing organizational capabilities and sustainability [16].

The World Health Organization’s global network of public health professionals, ExpandNet, proposed that scaling up involves “efforts to increase the impact of health service innovations successfully tested in pilot or experimental projects so as to benefit more people and to foster policy and program development on a lasting basis” [11]. This definition highlights the scientific or technical dimension (proven interventions), the political dimension, and the challenge of sustainability.

### Scaling-up domains

The 15 sources we reviewed differed in their approach to studying and explaining scale and the overall intent of the work (descriptive versus normative). Some included frameworks designed to assist program managers, implementers, and other actors in making practical decisions about scaling up. Some sources also provided insight into key capabilities or choices associated with success or failure in achieving scale. Despite the disparate nature of the sources studied, we were able to synthesize domains that consolidated much of the conceptual thinking about scale and scaling up found in the literature.

We identified 10 domains, each common to at least three of these works and included as integral to successful scaling up. While some of the domains were found across most of the reviewed publications, none were explicitly included in all 15. These domains were important to provide a framework from which to explore the next phase of project development: sustaining delivery at scale.

1. *Fiscal support*

Ensuring adequate [17], flexible [18], reliable, and sustainable funding [7,15,19]. This can be accomplished by incorporating a program into the national budget [8,11,20] or the core budget of the funding agency.

2. *Political support*

Mobilizing support for the program and protecting it from vested interests that may perceive it as a threat [9]. Obtaining the support of political leadership and champions who ensure sustained, visible, and high-level commitment to the program [17] at all levels of government and among relevant private-sector actors and civil society organizations [21].

3. *Community involvement, integration, buy-in, and depth*

Striking an appropriate balance between participatory and expert or management-dominated approaches [11]. Grounding scaling up in the principles of respect for and promotion of human rights and in the value of participatory and client-centered approaches [7]. Adapting the program to local contexts [11] and addressing the community’s identified needs [17]. End users should be engaged early on [11] and community champions involved in program design, implementation and scale-up [15]. Cultivating the depth of change necessary to support and sustain consequential change [14].

4. *Partnerships*

Ensuring that domestic and external partners either continue or are engaged to support the program [9]. Includes a systemic view of the variety of actors in the broader environment and a strategic understanding of how they can be leveraged to influence the scaling-up process [8]. Determining and ensuring appropriate balance of scaling-up responsibilities—additive (full burden on one organization) or multiplicative (distributed across several organizations) [11].

5. *Balancing flexibility/adaptability and standardization*

Striking an appropriate balance between flexible, adaptive strategies and implementing a standard package of interventions [11]. Ensuring that universally effective components of an intervention are applied while allowing for local adaptation [9]. Evaluating, learning, and changing the approach as scaling up proceeds and developing a culture of adaptation, flexibility, and openness to change [9]. Planning for context-specific delivery mechanisms effective in going to scale [22].

6. *Supportive policy, regulatory, and legal environment*

Ensuring that a supportive policy, regulatory, and legal framework [7,9,18] has been developed that allows for scaling up. Inclusion of program in national policies [7,8,11].

7. *Building and sustaining strong organizational capacity*

Addressing shortcomings in organizational capacity and enhancing the ability to deliver intended services and support [7,9]. May include building local capacity [17] and partnering with others able to operate the scaled program [9]. Ensuring staff is sufficient, well distributed, and qualified with strong technical and program management abilities [18]. Strengthening human capacities [11] in management and implementation within national and sub-national governments [17].

8. *Transferring ownership*

Shifting ownership so that it is no longer an “external” process controlled by reformers but instead becomes an “internal” process led by local actors with the capacity to sustain, spread, and deepen the results [14]. May include successfully transferring intervention to adopting organizations including to national or local government [9].

9. *Decentralization*

Determining and ensuring the appropriate balance of reach, influence, and resources provided by centralized authorities and local initiative, autonomy, spontaneity, mutual learning, and problem-solving provided by a decentralized approach [11]. Decentralizing management [17,18] and programmatic activities to the local level [17].

10. *Ongoing focus on sustainability*

Creating a lasting programmatic and policy impact that produces enduring health benefits [7,8,11]. Consistently focusing on sustainability [11] and devising a strategy that includes plans and actions to ensure sustainability. This focus may inform the path chosen to achieve scale. Uvin argues that this decision on path should reflect the nature of the intervention and local environment and may influence ability and need for sustainability [11].

The reviewed literature also identifies several other decision points in planning the scaling up of public health programs that are likely to inpact sustaining delivery at scale and reflect the complexity of replication of a program. These choices include decisions about when and if to transition to another organization (including the government) and the importance of fidelity to an effective model versus the latitude to adapt to local conditions [23].

### Sustaining delivery at scale domains: lessons learned from HIV/AIDS

While we found a general domain of an “ongoing focus on sustainability”, there were few references to how the other domains were relevant to sustaining delivery at scale. Our review of the 11 case studies showed each of the 10 domains had elements relevant to how these programs worked to sustain delivery at scale. Some of the domains became even more critical as programs transitioned from having achieved scale to aiming for sustaining delivery at scale. For example, for Domain 5 (*balancing flexibility/adaptability and standardization*), we found that as programs achieve scale, many struggle to balance continued local innovation with standardization under central control. The case studies suggest that program monitoring and evaluation and elements of financial control and oversight, as well as areas where there are economies of scale such as supply chain management and training, can be successfully standardized and contribute to sustainability. Elements of delivery models that will continue to need iterative adaptation include generating local demand and mobilizing communities.

The Avahan Indian AIDS Initiative, a large-scale HIV prevention program of the Bill and Melinda Gates Foundation, for example, used similar metrics and financial management structures across its seven “state lead partners” and 137 district-level NGOs that were implementing HIV prevention programs. But each NGO used a unique set of activities to achieve its goals and reach the local target population. Avahan achieved high levels of success using this model balancing management and monitoring with local adaptation of interventions to reflect targeted populations. To date, Avahan’s model is being evaluated as it transfers its model to the public sector. Further research is required to understand how and where flexibility ensures ongoing impact and where it poses a threat.

While Domain 7 (*building and sustaining strong organizational capacity*) was critical to sustaining delivery at scale, an even broader long-term approach to capacity was also found to be important. This approach should expand to include a focus on providers and key staff implementing the program by investing in long-term human resource retention and development. For example, a mature program might lose experienced staff if there is little room for professional development or “burnout” from prolonged time working in weak health systems and if there are ample opportunities to attain better-paying positions elsewhere. If programs do not plan for retention and staff turnover, there will be inadequate numbers of appropriately trained new staff (both internally for program management and for implementers as programs transfer to public sector), threatening sustainability of gains.

Therefore, a focus not only on meeting human resource demands as programs scale, but on developing internal human resources management to improve retention and maintenance of program expertise is likely to be essential for sustaining delivery at scale. Organizations featured in the case studies that have successfully sustained delivery at scale have deliberately created a culture of commitment and optimism among their employees to address staff retention [24,25]. The director of The AIDS Support Organization (TASO), a Ugandan NGO, for example, looked for motivated young people to hire and gave them significant responsibility. The antiretroviral therapy coordinator explained, “[Working at TASO] was very, very exciting, and people were so empowered with hope—'I can do this! This is work that was once restricted to doctors, but now I’m actually saving lives!’” The director valued good management skills over HIV knowledge in hiring and made sure staff members felt that they had a career advancement path within the organization.

The World Health Organization/ExpandNet framework includes equity as an important component in scaling up [11], but it was not prominent in other sources reviewed. As the vision for scaled programs is increasingly focused on transition to the public sector as the main pathway to sustainability, an emphasis on equity (i.e., a focus on ensuring access that includes the most vulnerable) needs to be an increasingly important driver of decisions and delivery models to ensure that this sector is able to fulfill the role of serving the entire population [26]. Iran’s Triangular Clinic, for example, served HIV-positive individuals from various risk groups, many of whom were injection drug users who had been ostracized from their families and society at large. Assisting all populations affected by HIV, not just those easiest to reach or least stigmatized, initially led to a profound local impact and was later adopted by the national program, scaled up around the country, and integrated with the health care system. We would therefore propose an 11th domain: emphasizing equity.

## Discussion

While there was heterogeneity in the focus and format of our sources, we were able to identify 10 domains commonly considered integral for scaling up (see Table 3 for domains). We also found that all 10 were relevant to the case study programs that were focused on sustaining delivery after achieving scale, although some domains may need to be expanded depending on the definitions used. Addressing all 10 of these domains may not be necessary for successfully sustaining delivery at scale of all program types. Instead, it is likely that the relative importance will differ depending on the sector where scale occurred, type of program, and local and national factors. Conversely, fulfilling all 10 domains may not guarantee success in sustaining delivery and effectiveness at scale. For example, few of the sources explicitly included effectiveness or quality as central to their frameworks.

**Table 3:** Ten domains relevant to scaling up extracted from the literature review

**Domain**	**Definition(s) from primary sources**
Fiscal support	Ensuring adequate, flexible, reliable, and sustainable funding. This can be accomplished by incorporating a program into the national budget or the core budget of the funding agency.
Political support	Mobilizing support for the program and protecting it from vested interests that may perceive it as a threat. Obtaining the support of political leadership and champions who ensure sustained, visible, and high-level commitment to the program at all levels of government and among relevant private-sector actors and civil society organizations.
Community involvement, integration, buy-in, and depth	Striking an appropriate balance between participatory and expert or management-dominated approaches. Grounding scaling up in the principles of respect for and promotion of human rights and in the value of participatory and client-centered approaches. Adapting the program to local contexts and addressing the community’s identified needs. End users should be engaged early on and community champions involved in program design, implementation, and scale-up. Cultivating the depth of change necessary to support and sustain consequential change.
Partnerships	Ensuring that domestic and external partners either continue or are engaged to support the program. Includes a systemic view of the variety of actors in the broader environment and a strategic understanding of how they can be leveraged to influence the scaling-up process. Determining and ensuring appropriate balance of scaling-up responsibilities—additive (full burden on one organization) or multiplicative (distributed across several organizations).
Balancing flexibility/adaptability and standardization	Striking an appropriate balance between flexible, adaptive strategies and implementing a standard package of interventions. Ensuring that universally effective components of an intervention are applied while allowing for local adaptation. Evaluating, learning, and changing the approach as scaling up proceeds and developing a culture of adaptation, flexibility, and openness to change. Planning for context-specific delivery mechanisms effective in going to scale.
Supportive policy, regulatory, and legal environment	Ensuring that a supportive policy, regulatory, and legal framework has been developed that allows for operating at scale. Inclusion of program in national policies.
Building and sustaining strong organizational capacity	Addressing shortcomings in organizational capacity and enhancing the ability to deliver intended services and support. May include building local capacity and partnering with others able to operate the scaled program. Ensuring staff is sufficient, well distributed, and qualified with strong technical and program management abilities. Strengthening human capacities in management and implementation within national and sub-national governments.
Transferring ownership	Shifting ownership so that it is no longer an “external” process controlled by reformers but instead becomes an “internal” process led by local actors with the capacity to sustain, spread, and deepen the results. May include successfully transferring intervention to adopting organizations.
Decentralization	Determining and ensuring the appropriate balance of reach, influence, and resources provided by centralized authorities and local initiative, autonomy, spontaneity, mutual learning, and problem-solving provided by a decentralized approach. Decentralizing management and programmatic activities to the local level.
Ongoing focus on sustainability	Creating a lasting programmatic and policy impact that produces enduring health benefits. Consistently focusing on sustainability and devising a strategy that includes plans and actions to ensure sustainability. This focus may inform the path chosen to achieve scale.

Hartmann and Linn identify the notion of a “learning space” that involves building a culture of innovation as important for achieving scale [9]. It is likely such a space will be important for sustaining scale as well. HIV programs trying to sustain delivery at scale will need to consider how they achieved scale—previous choices made and forces involved—designing their strategies and planning for moving forward. The ability to achieve and to maintain scale likely will be enhanced by deliberately planning, monitoring progress, and adapting to accommodate findings.

We believe the applicability of the 10 domains identified from the scaling-up literature should be more formally assessed to guide HIV programs focused on sustaining delivery at scale. For example, it is likely the tactics of program management will change with the transition from scaling up to sustaining, but the need for management capacity will remain. Therefore, underfunding management capacity, identified as important in scaling and from the case studies in sustaining delivery at scale, may threaten sustainability. Another example is the need to continue or increase focus on quality in programs operating at scale. This may be particularly true for HIV prevention and treatment programs that were established and scaled up quickly to meet the treatment gap. As some of these programs scaled up, they emphasized coverage. For example, Avahan did not document core program elements or define a set of operating standards for its first two years while it recruited its community-level partners and implementers, primarily focusing on implementing. After two years, the Common Minimum Program was introduced, which codified existing innovations from the field, including those related to community mobilization and clinical services for the first time, and created a means to define quality as well as programmatic targets.

Some programs we reviewed also began with limited focus on engagement with and strengthening of the public sector, a potential barrier to sustaining delivery at scale. The case of loveLife demonstrates this well. While loveLife began operations in 1999, it wasn’t until 2005 when it lost over one-third of its funding, which had come from the private sector, that it really began thinking about how to engage the public sector and increase government participation. By 2008, the South African government provided 75% of loveLife’s revenue, marking a nearly complete transition from international donor funding to domestic government funding and success in sustaining delivery at scale.

Similar to the complexities of defining scale and the many dimensions of scale up described by Uvin, identifying core domains needed for sustainability will require a multifaceted and systems-focused approach. The conceptualization of sustainability will need to go beyond coverage and encompass the need for ongoing work to support the other facets of scale, including depth of programmatic capacity and community engagement [27]. Such dimensions are likely to be critical in allowing a program to adapt and continue to generate maximum value for populations.

There are a number of limitations in this paper. The 15 sources we used to extract the domains were heterogeneous, using varying units of analysis. In addition, we did not try to do an exhaustive extraction of all resources on scaling up and scale in public health. We also did not “weight” the domains based on the number of times they were referenced, relying on a qualitative approach focused on identifying cross-cutting main themes. We also did not include scaled programs that failed and did not formally map the 10 domains derived from scaling up and the additional one proposed for sustaining delivery at scale against HIV programs other than the case studies.

Our decision to focus on HIV/AIDS potentially limited the generalizability of our conclusions. In addition, the cases reviewed did not focus on the impact of changes in HIV related care and treatment (such as simplification of regimens and point-of-care testing), so we could not identify the effects of adoption of advances in technology and treatment on success for scale-up or sustaining delivery at scale. Finally, we did not assess the outcomes produced by the programs at scale. Although based on the expert consultations, there was general consensus that these were exemplar programs of scale, we did not look at domains needed to determine which programs actually should be scaled. Considering the current limited resources and demand for effectiveness and efficiencies in health care delivery, guidance is needed for funders to determine and project how to sustain delivery at scale [27].

## Conclusions

Numerous scaling up frameworks and conceptual models exist to help implementers plan programs and take pilots to scale. However, there is a relative dearth of literature and tools available for implementers, donors, and governments focused on sustaining scaled programs. We propose using 10 domains from scaling up and one additional domain, emphasizing equity, to start addressing this gap, but further work is needed. Once developed, a framework could serve as guidance on how to continue to realize the promise of HIV programs by helping stakeholders sustain programs that are successful at generating health for populations.

## Endnote

^a^To see the Cases in Global Health Delivery, please visit http://www.ghdonline.org/cases or http://hbsp.harvard.edu/list/ghd.

## Competing interests

There are no competing interests to declare.

## Authors’ contributions

LRH participated in the conception and design of the study, analysis and interpretation of the data, and drafting/revising the paper. LRH is a physician whose research focuses on monitoring, evaluating, and improving access, utilization, and outcomes of HIV and primary care in resource limited settings; and adherence in people living with HIV. JRT participated in the analysis and interpretation of the data, drafting the paper, and revising it critically for substantial intellectual content. JRT is a public health researcher and writer who has participated extensively in documenting care delivery programs, including those focused on scaling up and sustaining delivery at scale. ACI participated in the conception and design of the study, analysis and interpretation of the data, and drafting/revising the paper. ACI has co-authored numerous books, including *Global AIDS: Myths and Facts.* MAM participated in the conception and design of the study, analysis and interpretation of the data, and drafting/revising the paper. MAM helped author several case studies on HIV programs. ND participated in the analysis and interpretation of the data, drafting the paper. She worked as a Senior Analyst for the Global Health Delivery Project. RS participated in the analysis and interpretation of the data, drafting the paper, and revising it critically for substantial intellectual content. RS worked on the GHD project and is working in Health Systems analysis. ALE participated in the review and summary of case studies describing organizations operating at scale, the identification and refinement of the domains described in the paper, and the writing of the results section. ALE is a physician whose research focuses on health systems improvements for underserved populations. RLW participated in the conception and design of the study, analysis and interpretation of the data, and drafting/revising the paper. RlW is a physician and the Faculty Director of the Global Health Delivery project whose research focuses on how health care delivery generates value for patients and populations. All authors read and approved the final manuscript.
